# (*R*)-2-Methyl-5-[(*R*)-2,4,4,4-tetra­chloro­butan-2-yl]cyclo­hex-2-enone

**DOI:** 10.1107/S1600536812031194

**Published:** 2012-07-14

**Authors:** Brahim Boualy, Mohamed Anouar Harrad, Larbi El Firdoussi, Mustapha Ait Ali, Helen Stoeckli-Evans

**Affiliations:** a Equipe de Chimie de Coordination, Faculté des Sciences Semlalia, BP 2390, Marrakech, Morocco; b Institute of Physics, University of Neuchâtel, 2000 Neuchâtel, Switzerland

## Abstract

The title compound, C_11_H_14_Cl_4_O, was efficiently synthesized by atom-transfer radical addition between (*R*)-carvone and tetra­chloro­methane. In the mol­ecule, both chiral centres are of the absolute configuration *R*. The cyclo­hex-2-enone ring has an envelope conformation with the chiral C atom displaced by 0.633 (2) Å from the mean plane through the other five C atoms [maximum deviation = 0.036 (2) Å]. In the crystal, mol­ecules are linked *via* C—H⋯O inter­actions, leading to the formation of helical chains propagating along [100].

## Related literature

For synthetic details, see: Boualy *et al.* (2011 ▶); Dragutan *et al.* (2007 ▶). For related structures, see: Boualy *et al.* (2009 ▶, 2011 ▶); Ziyat *et al.* (2004 ▶, 2006 ▶). For the distribution of caraway (*Carum carvi L*.), see: Carvalho da & Fonseca da (2006 ▶); Hornok (1992 ▶). For biological activity, see: Farag *et al.* (1989 ▶); Juaristi & Soloschonok (2005 ▶); Nagashima *et al.* (2003 ▶); Reynolds (1987 ▶); Saxena *et al.* (1987 ▶); Zheng *et al.* (1992 ▶). For carvone derivatives having olfactory properties, see: Buch & Wuest (1969 ▶); Aurrecoechea & Okamura (1987 ▶); Torii *et al.* (1983 ▶).
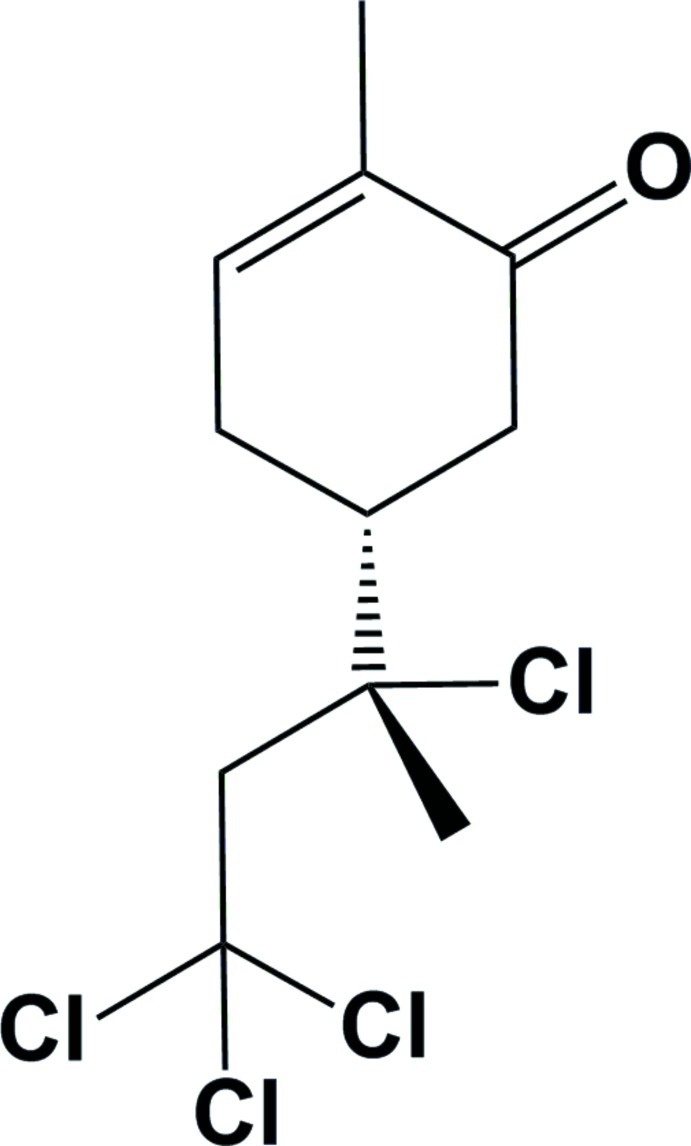


## Experimental

### 

#### Crystal data

C_11_H_14_Cl_4_O
*M* *_r_* = 304.02
Orthorhombic, 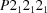

*a* = 6.4976 (6) Å
*b* = 13.3343 (16) Å
*c* = 15.7648 (14) Å
*V* = 1365.9 (2) Å^3^
*Z* = 4
Mo *K*α radiation
μ = 0.84 mm^−1^
*T* = 293 K
0.38 × 0.27 × 0.20 mm

#### Data collection

Stoe IPDS diffractometer
Absorption correction: multi-scan (*MULscanABS* in *PLATON*; Spek, 2009 ▶) *T*_min_ = 0.963, *T*_max_ = 1.000
9786 measured reflections
2431 independent reflections
1662 reflections with *I* > 2σ(*I*)
*R*_int_ = 0.033

#### Refinement

*R*[*F* ^2^ > 2σ(*F* ^2^)] = 0.029
*wR*(*F* ^2^) = 0.063
*S* = 0.87
2431 reflections
148 parameters
H-atom parameters constrained
Δρ_max_ = 0.21 e Å^−3^
Δρ_min_ = −0.19 e Å^−3^
Absolute structure: Flack (1983 ▶), 1005 Friedel pairs
Flack parameter: 0.00 (7)

### 

Data collection: *EXPOSE* in *IPDS*-I (Stoe & Cie, 2004 ▶); cell refinement: *CELL* in *IPDS*-I (Stoe & Cie, 2004 ▶); data reduction: *INTEGRATE* in *IPDS*-I (Stoe & Cie, 2004 ▶); program(s) used to solve structure: *SHELXS97* (Sheldrick, 2008 ▶); program(s) used to refine structure: *SHELXL97* (Sheldrick, 2008 ▶); molecular graphics: *PLATON* (Spek, 2009 ▶) and *Mercury* (Macrae *et al.*, 2008 ▶); software used to prepare material for publication: *SHELXL97*, *PLATON* and *publCIF* (Westrip, 2010 ▶).

## Supplementary Material

Crystal structure: contains datablock(s) I, global. DOI: 10.1107/S1600536812031194/kp2431sup1.cif

Structure factors: contains datablock(s) I. DOI: 10.1107/S1600536812031194/kp2431Isup2.hkl

Supplementary material file. DOI: 10.1107/S1600536812031194/kp2431Isup3.cml

Additional supplementary materials:  crystallographic information; 3D view; checkCIF report

## Figures and Tables

**Table 1:** Hydrogen-bond geometry (Å, °)

*D*—H⋯*A*	*D*—H	H⋯*A*	*D*⋯*A*	*D*—H⋯*A*
C8—H8*A*⋯O1^i^	0.97	2.51	3.394 (3)	152

Symmetry code: (i) 
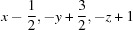
.

**Fig. 1.**
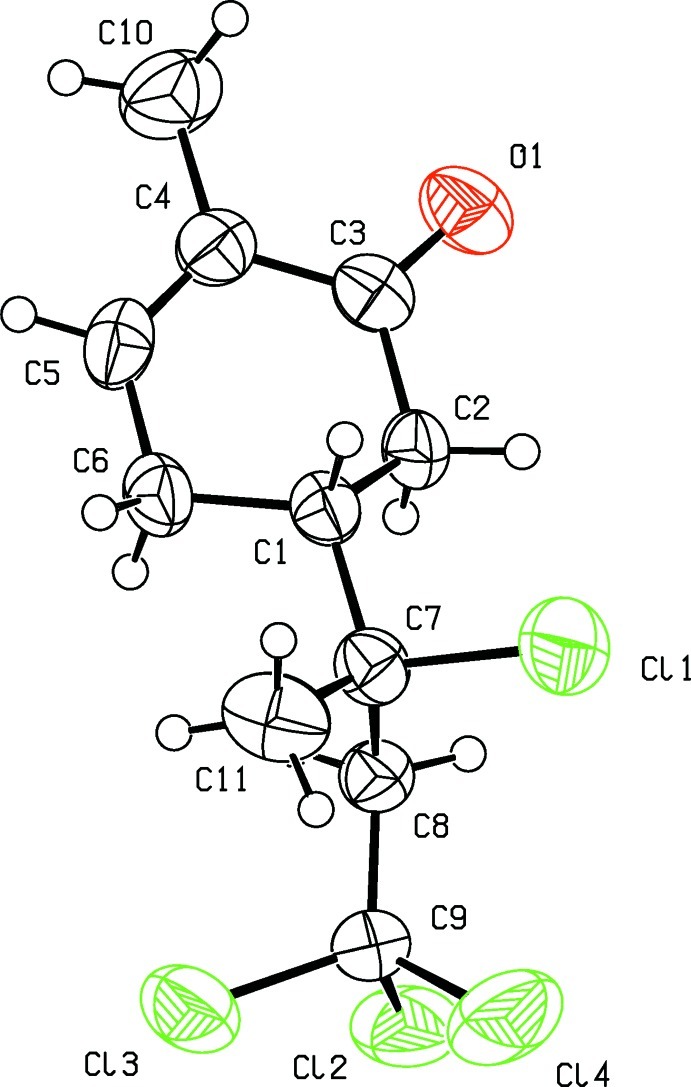
The molecular structure of the title molecule, showing the crystallographic atom numbering scheme. The displacement ellipsoids are drawn at the 50% probability level.

**Fig. 2.**
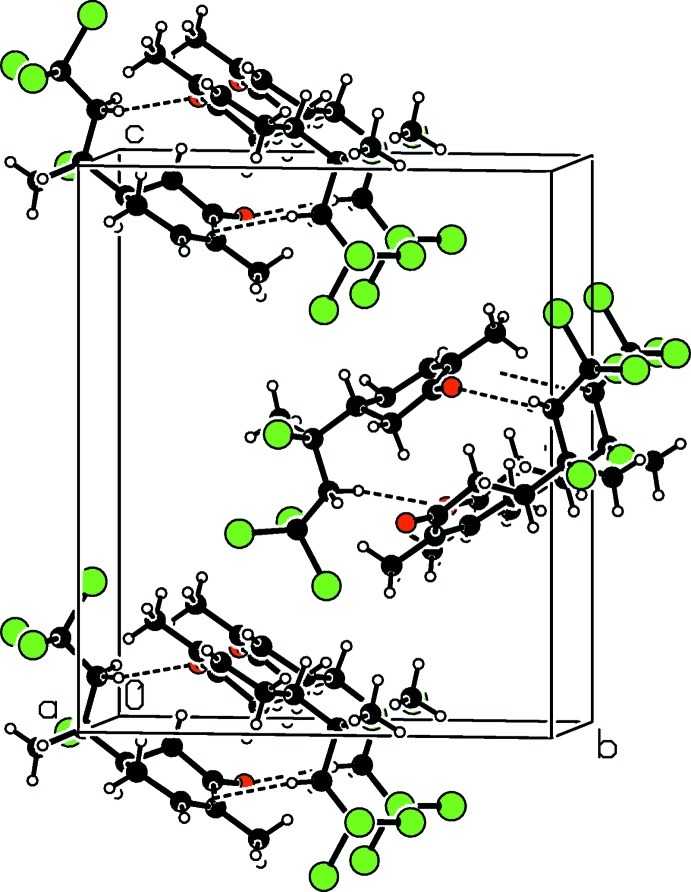
A view along the a axis of the crystal packing of the title compound, showing the C—H···O interactions (dashed lines) leading to the formation of helical chains propagating along [100].

**Fig. 3.**
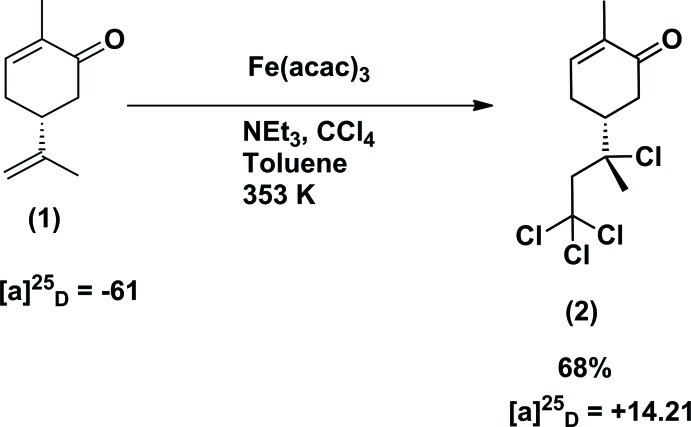
Synthesis of the title compound (Boualy et al., 2011).

## supplementary crystallographic information

### Comment

Caraway (Carum carvi *L*.) is naturally found in Northern and Central Europe, Siberia, Turkey, Iran, India and North Africa (Carvalho da & Fonseca da, 2006). The main constituent (50–70%) of the essential Caraway oil is (4*S*)-(+)-carvone (Hornok, 1992). This monoterpene exhibits some interesting biological activities, such as antimicrobial (Farag *et al.*, 1989), nematicidal (Saxena *et al.*, 1987), antitumor (Zheng *et al.*, 1992) and plant growth regulatory activities (Reynolds, 1987). In fact, many syntheses from carvone were reported in order to prepare new compounds having olfactory properties suitable in various fields (Buch & Wuest, 1969; Aurrecoechea & Okamura, 1987; Torii *et al.*, 1983).

The Kharasch addition or atom transfer radical addition (ATRA) is a synthetically useful process for functionalizing organic compounds by means of halogen derivatives (Dragutan *et al.*, 2007). They are applied in the synthesis of polyfunctional acyclic and heterocyclic compounds, such as β-aminoacids (Juaristi & Soloschonok, 2005) and alkaloids (Nagashima *et al.*, 2003). As a part of our interest in the synthesis of optically actives polyhalogenated products from terpenes (Boualy *et al.*, 2009; Ziyat *et al.*, 2004; Ziyat *et al.*, 2006; Boualy *et al.*, 2011) we report herein on the synthesis and crystal structure of the title compound. It is a new polyhalogenated terpene from (*R*)-carvone, which could be a valuable precursor for the synthesis of new polyfunctional terpenic compounds.

The title compound (Fig. 1) was obtained as a colourless solid by addition of tetrachloromethane to (*R*)-carvone catalyzed by Fe(acac)_3_ in toluene at 353 K (Boualy *et al.*, 2011). The two chiral centres, C1 and C7, have *R* absolute configurations (Fig. 1). The cyclohex-2-enone ring (C1—C6) has an envelope conformation with the chiral C atom, C1, displaced by 0.633 (2) Å from the mean plane through the other five C atoms [C2—C6; maximum deviation 0.036 (2) Å]. In the molecule there is a short C2—H2A···Cl1 contact (H2a···Cl1 = 2.72 Å; C2···Cl1 = 3.167 (2) Å).

In the crystal, C—H···O interactions (Table 1 and Fig. 2) are present and lead to the formation of helical chains propagating along [100].

The structure of the title molecule was also characterized by ^1^H, ^13^C NMR spectroscopy and by mass spectroscopy. ^1^H NMR data of the isolated product indicated the presence of an olefinic proton at 6.77 p.p.m. corresponding to the group (C═CH), which indicated also the absence of the olefinic protons of the group (C═CH_2_). In the ^13^C NMR spectrum, the signal of the carbonyl group was observed at δ = 199 p.p.m., the olefinic carbons appeared at 144.5 and 135.5 p.p.m. and the quaternary carbon containing three chlorine atoms appeared at 98.4 p.p.m. The conservation of the carbonyl group was also confirmed by the IR absorption at 1720 cm^-1^. The mass spectrum of the compound confirmed the proposed structure exhibiting a molecular ion peak at m/*z* 304 and the base peak at m/*z* 136, which originated from the monoterpene fragment.

The X-ray single-crystal analysis of the title compound clearly shows the absolute configuration at atoms C1 and C7 to be (*R*, *R*)(Fig.1).

### Experimental

The synthesis of the title compound (Boualy *et al.*, 2011) is illustrated in Fig. 3. A mixture of Fe(acac)_3_ (4.87 mg, 0.0138 mmol), NEt_3_ (4.485 mg, 0.044 mmol), (*R*)-Carvone (207.30 mg, 1.38 mmol) and CCl_4_ (849.09 mg, 5.52 mmol), was stirred in 353 K in 5 mL of toluene for 6 h and then hydrolyzed by addition of 20 mL of water. The organic layer was separated and the aqueous layer was washed with 3 × 10 mL of dichloromethane. The combined organic extracts were dried over Na_2_SO_4_ and concentred in a rotary evaporator at reduced pressure. Column chromatography (hexane / ethyl acetate: 5:1 *v*/*v*) of the residue on silica gel gave 285.76 mg (0.94 mmol, 68%) of the title compound as a colourless solid. Recrystallization in chloroform at rt afforded colourless rod-like crystals suitable for X-ray crystallographic analysis. *M*. p. 411 K; [α]^20^_D_ = +14.2 (c = 1.01, CHCl_3_); ^1^H NMR (300 MHz, CDCl_3_): δ = 1.70 (S, 3H); 1.85 (S, 3H), 2.24–2.67 (m, 5H), 3.35 (S, éH), 6.50 (m, 1H); ^13^C NMR (75 MHz, CDCl_3_): δ = 15.55 (CH_3_–), 27.37 (CH_3_–), 27.89 (–CH_2_–), 39.83 (–CH_2_–), 45.74 (–CH–), 62.50 (–CH_2_—CCl_3_), 73.31 (–C—Cl), 96.15 (–CCl_3_), 135.68 (=Cq), 142.52 (=CH), 197.26 (C=O); MS (EI, 70 eV): m/*z* (%) = 304 [*M*+].

### Refinement

C-bound H-atoms were included in calculated positions and treated as riding atoms: C—H = 0.93, 0.98, 0.97 and 0.96 Å for CH(allyl), CH(methine), CH_2_, and CH_3_ H-atoms, respectively, with *U*_iso_(H) = k × *U*_eq_(C), where k = 1.5 for CH_3_ H-atoms and = 1.2 for other H-atoms.

### Crystal data

**Table:**

C_11_H_14_Cl_4_O	*F*(000) = 624
*M**_r_* = 304.02	*D*_x_ = 1.478 Mg m^−^^3^
Orthorhombic, *P*2_1_2_1_2_1_	Mo *K*α radiation, λ = 0.71073 Å
Hall symbol: P 2ac 2ab	Cell parameters from 6130 reflections
*a* = 6.4976 (6) Å	θ = 2.5–21.9°
*b* = 13.3343 (16) Å	µ = 0.84 mm^−^^1^
*c* = 15.7648 (14) Å	*T* = 293 K
*V* = 1365.9 (2) Å^3^	Rod, colourless
*Z* = 4	0.38 × 0.27 × 0.20 mm

### Data collection

**Table:**

Stoe IPDS diffractometer	2431 independent reflections
Radiation source: fine-focus sealed tube	1662 reflections with *I* > 2σ(*I*)
Graphite monochromator	*R*_int_ = 0.033
φ rotation scans	θ_max_ = 25.1°, θ_min_ = 2.6°
Absorption correction: multi-scan (MULscanABS in *PLATON*; Spek, 2009)	*h* = −7→7
*T*_min_ = 0.963, *T*_max_ = 1.000	*k* = −15→15
9786 measured reflections	*l* = −18→18

### Refinement

**Table:**

Refinement on *F*^2^	Secondary atom site location: difference Fourier map
Least-squares matrix: full	Hydrogen site location: inferred from neighbouring sites
*R*[*F*^2^ > 2σ(*F*^2^)] = 0.029	H-atom parameters constrained
*wR*(*F*^2^) = 0.063	*w* = 1/[σ^2^(*F*_o_^2^) + (0.0338*P*)^2^] where *P* = (*F*_o_^2^ + 2*F*_c_^2^)/3
*S* = 0.87	(Δ/σ)_max_ < 0.001
2431 reflections	Δρ_max_ = 0.21 e Å^−^^3^
148 parameters	Δρ_min_ = −0.19 e Å^−^^3^
0 restraints	Absolute structure: Flack (1983), 1005 Friedel pairs
Primary atom site location: structure-invariant direct methods	Flack parameter: 0.00 (7)

### Special details

**Table:**

Geometry. Bond distances, angles *etc*. have been calculated using the rounded fractional coordinates. All su's are estimated from the variances of the (full) variance-covariance matrix. The cell e.s.d.'s are taken into account in the estimation of distances, angles and torsion angles

### Fractional atomic coordinates and isotropic or equivalent isotropic displacement parameters (Å^2^)

**Table:**

	*x*	*y*	*z*	*U*_iso_*/*U*_eq_	
Cl1	0.83987 (14)	0.40824 (6)	0.52522 (6)	0.0812 (3)	
Cl2	0.52828 (15)	0.48377 (7)	0.24964 (5)	0.0845 (3)	
Cl3	0.23706 (13)	0.38341 (7)	0.35431 (5)	0.0834 (3)	
Cl4	0.65044 (18)	0.31020 (7)	0.34378 (6)	0.1061 (4)	
O1	0.8569 (3)	0.77674 (13)	0.61294 (12)	0.0671 (7)	
C1	0.5690 (4)	0.55138 (18)	0.56903 (14)	0.0423 (8)	
C2	0.7126 (4)	0.63646 (17)	0.54387 (15)	0.0474 (9)	
C3	0.7060 (4)	0.72331 (18)	0.60355 (14)	0.0471 (9)	
C4	0.5098 (4)	0.74598 (18)	0.64494 (14)	0.0502 (9)	
C5	0.3492 (5)	0.6861 (2)	0.63074 (14)	0.0545 (10)	
C6	0.3522 (4)	0.59286 (19)	0.57915 (15)	0.0518 (9)	
C7	0.5809 (4)	0.45906 (18)	0.51053 (15)	0.0474 (9)	
C8	0.5589 (4)	0.49132 (17)	0.41742 (13)	0.0468 (9)	
C9	0.4989 (4)	0.41885 (19)	0.34799 (16)	0.0561 (9)	
C10	0.4985 (6)	0.8381 (2)	0.69942 (18)	0.0788 (14)	
C11	0.4329 (5)	0.3768 (2)	0.54069 (17)	0.0696 (10)	
H1	0.61290	0.52900	0.62540	0.0510*	
H2A	0.85230	0.61100	0.54130	0.0570*	
H2B	0.67570	0.65950	0.48750	0.0570*	
H5	0.22450	0.70430	0.65520	0.0650*	
H6A	0.29500	0.60680	0.52350	0.0620*	
H6B	0.26610	0.54270	0.60610	0.0620*	
H8A	0.45870	0.54520	0.41630	0.0560*	
H8B	0.68970	0.52050	0.40090	0.0560*	
H10A	0.52610	0.89630	0.66540	0.1180*	
H10B	0.59880	0.83350	0.74400	0.1180*	
H10C	0.36350	0.84340	0.72370	0.1180*	
H11A	0.45650	0.36340	0.59970	0.1050*	
H11B	0.45610	0.31680	0.50840	0.1050*	
H11C	0.29350	0.39880	0.53280	0.1050*	

### Atomic displacement parameters (Å^2^)

**Table:**

	*U*^11^	*U*^22^	*U*^33^	*U*^12^	*U*^13^	*U*^23^
Cl1	0.0681 (6)	0.0751 (5)	0.1004 (6)	0.0255 (5)	−0.0196 (5)	−0.0134 (4)
Cl2	0.0987 (7)	0.1091 (7)	0.0457 (3)	−0.0243 (5)	0.0049 (4)	−0.0112 (4)
Cl3	0.0705 (6)	0.1037 (6)	0.0760 (5)	−0.0304 (4)	−0.0028 (5)	−0.0140 (5)
Cl4	0.1214 (8)	0.0882 (6)	0.1088 (7)	0.0334 (6)	−0.0023 (6)	−0.0402 (6)
O1	0.0625 (14)	0.0576 (11)	0.0812 (12)	−0.0165 (11)	−0.0107 (11)	−0.0027 (9)
C1	0.0386 (17)	0.0511 (14)	0.0371 (12)	−0.0032 (12)	−0.0011 (10)	0.0052 (10)
C2	0.0364 (16)	0.0561 (15)	0.0498 (14)	−0.0051 (12)	0.0052 (12)	−0.0003 (12)
C3	0.0510 (19)	0.0434 (14)	0.0469 (13)	−0.0009 (13)	−0.0095 (13)	0.0082 (11)
C4	0.0541 (18)	0.0535 (15)	0.0429 (12)	0.0091 (14)	−0.0050 (14)	0.0011 (12)
C5	0.0459 (18)	0.0702 (17)	0.0473 (15)	0.0167 (15)	0.0057 (13)	0.0083 (13)
C6	0.0377 (16)	0.0712 (17)	0.0464 (14)	−0.0053 (15)	0.0029 (12)	0.0042 (12)
C7	0.0454 (18)	0.0475 (14)	0.0493 (13)	0.0012 (12)	−0.0038 (11)	0.0022 (11)
C8	0.0461 (17)	0.0507 (15)	0.0436 (12)	−0.0038 (13)	0.0023 (12)	−0.0030 (11)
C9	0.0516 (18)	0.0648 (16)	0.0519 (14)	−0.0021 (14)	0.0015 (14)	−0.0167 (13)
C10	0.093 (3)	0.066 (2)	0.0774 (19)	0.0220 (19)	−0.0065 (19)	−0.0139 (16)
C11	0.089 (2)	0.0573 (16)	0.0624 (16)	−0.0234 (16)	−0.0038 (17)	0.0118 (14)

### Geometric parameters (Å, º)

**Table:**

Cl1—C7	1.829 (3)	C8—C9	1.511 (3)
Cl2—C9	1.786 (3)	C1—H1	0.9800
Cl3—C9	1.769 (3)	C2—H2A	0.9700
Cl4—C9	1.753 (3)	C2—H2B	0.9700
O1—C3	1.221 (3)	C5—H5	0.9300
C1—C2	1.522 (3)	C6—H6A	0.9700
C1—C6	1.522 (4)	C6—H6B	0.9700
C1—C7	1.540 (3)	C8—H8A	0.9700
C2—C3	1.493 (3)	C8—H8B	0.9700
C3—C4	1.464 (4)	C10—H10A	0.9600
C4—C5	1.333 (4)	C10—H10B	0.9600
C4—C10	1.501 (4)	C10—H10C	0.9600
C5—C6	1.486 (4)	C11—H11A	0.9600
C7—C8	1.536 (3)	C11—H11B	0.9600
C7—C11	1.534 (4)	C11—H11C	0.9600
			
C2—C1—C6	108.9 (2)	C1—C2—H2B	109.00
C2—C1—C7	114.2 (2)	C3—C2—H2A	109.00
C6—C1—C7	113.6 (2)	C3—C2—H2B	109.00
C1—C2—C3	113.4 (2)	H2A—C2—H2B	108.00
O1—C3—C2	120.4 (2)	C4—C5—H5	117.00
O1—C3—C4	121.7 (2)	C6—C5—H5	117.00
C2—C3—C4	117.8 (2)	C1—C6—H6A	109.00
C3—C4—C5	118.9 (2)	C1—C6—H6B	109.00
C3—C4—C10	117.8 (2)	C5—C6—H6A	109.00
C5—C4—C10	123.2 (3)	C5—C6—H6B	109.00
C4—C5—C6	125.7 (3)	H6A—C6—H6B	108.00
C1—C6—C5	111.9 (2)	C7—C8—H8A	107.00
Cl1—C7—C1	105.46 (17)	C7—C8—H8B	107.00
Cl1—C7—C8	108.08 (17)	C9—C8—H8A	107.00
Cl1—C7—C11	105.82 (18)	C9—C8—H8B	107.00
C1—C7—C8	110.10 (19)	H8A—C8—H8B	107.00
C1—C7—C11	110.8 (2)	C4—C10—H10A	109.00
C8—C7—C11	116.0 (2)	C4—C10—H10B	109.00
C7—C8—C9	122.5 (2)	C4—C10—H10C	109.00
Cl2—C9—Cl3	106.34 (14)	H10A—C10—H10B	109.00
Cl2—C9—Cl4	107.92 (14)	H10A—C10—H10C	110.00
Cl2—C9—C8	106.93 (17)	H10B—C10—H10C	109.00
Cl3—C9—Cl4	108.76 (14)	C7—C11—H11A	109.00
Cl3—C9—C8	112.22 (18)	C7—C11—H11B	109.00
Cl4—C9—C8	114.27 (18)	C7—C11—H11C	109.00
C2—C1—H1	107.00	H11A—C11—H11B	109.00
C6—C1—H1	107.00	H11A—C11—H11C	109.00
C7—C1—H1	107.00	H11B—C11—H11C	110.00
C1—C2—H2A	109.00		
			
C6—C1—C2—C3	54.6 (3)	O1—C3—C4—C10	0.4 (3)
C7—C1—C2—C3	−177.3 (2)	C2—C3—C4—C5	3.0 (3)
C2—C1—C6—C5	−47.4 (3)	C2—C3—C4—C10	−175.2 (2)
C7—C1—C6—C5	−175.77 (19)	C3—C4—C5—C6	3.5 (4)
C2—C1—C7—Cl1	65.6 (2)	C10—C4—C5—C6	−178.5 (2)
C2—C1—C7—C8	−50.8 (3)	C4—C5—C6—C1	20.2 (3)
C2—C1—C7—C11	179.6 (2)	Cl1—C7—C8—C9	84.7 (3)
C6—C1—C7—Cl1	−168.79 (16)	C1—C7—C8—C9	−160.6 (2)
C6—C1—C7—C8	74.8 (3)	C11—C7—C8—C9	−33.9 (3)
C6—C1—C7—C11	−54.8 (3)	C7—C8—C9—Cl2	−171.8 (2)
C1—C2—C3—O1	151.4 (2)	C7—C8—C9—Cl3	71.9 (3)
C1—C2—C3—C4	−33.0 (3)	C7—C8—C9—Cl4	−52.5 (3)
O1—C3—C4—C5	178.5 (2)		

### Hydrogen-bond geometry (Å, º)

**Table:**

*D*—H···*A*	*D*—H	H···*A*	*D*···*A*	*D*—H···*A*
C8—H8*A*···O1^i^	0.97	2.51	3.394 (3)	152

Symmetry code: (i) *x*−1/2, −*y*+3/2, −*z*+1.
